# A systematic review of stereotactic radiosurgery for pituitary metastases

**DOI:** 10.1007/s11060-026-05424-7

**Published:** 2026-01-21

**Authors:** Trent Kite, Nikitha Harikumar, Stephen Jaffee, Henry Knox, John Herbst, Stephen Karlovits, Alexander Yu, Jody Leonardo, Rodney E. Wegner, Matthew J. Shepard

**Affiliations:** 1 Department of Neurosurgery, Allegheny Health Network, Neuroscience Institute, 320 E North Ave, Pittsburgh, PA 15212 USA; 2 Drexel University College of Medicine, Philadelphia, PA USA; 3 Edward Via College of Osteopathic Medicine, Spartanburg, SC USA; 4 Division of Oncology, Allegheny Health Network Cancer Institute, Pittsburgh, PA USA; 5 Division of Radiation Oncology, Allegheny Health Network Cancer Institute, Pittsburgh, PA USA

**Keywords:** Pituitary, Metastases, Stereotactic radiosurgery, Gamma knife, Cyberknife

## Abstract

**Background:**

Pituitary gland metastases (PGM) are extremely rare, and their ideal management is poorly defined. Radiosurgery for PGM may obviate the need for tumor excision, decreasing surgical morbidity in a patient population with an otherwise life-limiting illness. Given this, we sought to summarize existing literature on outcomes of PGM treated with stereotactic radiosurgery (SRS).

**Methods:**

A systematic review was conducted in accordance with the Preferred Reporting Items for Systematic Reviews and Meta-analyses (PRISMA). PubMed and Science Direct databases were queried from their inception through November 2025. The search phrase “((Stereotactic radiosurgery OR SRS OR radiosurgery) AND (Pituitary metastases))” was applied without any search filters. The primary outcome of interest was local tumor control (LC) with an aggregate local tumor control (LC) point estimate derived by calculating a weighted average across selected studies. Additional outcomes of interest were, post-SRS endocrinological function, and cranial nerve deficits.

**Results:**

Eight studies encompassing 119 patients with PGM treated with SRS were reviewed. A median tumor volume and diameter of 2.35 cm^3^, and 2.2 cm respectively was treated with a median prescription dose of 13.0 Gy. The dose delivered to the optic apparatus was <10 Gy in 85.7% of studies. The pooled local control rate following SRS was 90% (95% CI: 75%-99%) over a median follow up of 7 months. A pooled median survival estimate of 14 months (range: 5.2-30.0) was demonstrated. Diabetes insipidus, hypopituitarism, and cranial nerve deficits either improved or were stable following SRS in 50.0%, 53.6%, and 75.0% of patients respectively.

**Conclusion:**

SRS affords high LC in the setting of PGM. Despite this, survival is poor and likely depends on systemic disease control. A multidisciplinary approach incorporating SRS represents a promising option for this patient population. Further characterization of optimal radiosurgical parameters is warranted.

## Introduction

Pituitary gland metastasis (PGM) is a rare phenomenon in neuro-oncology [1–4]. In the context of improved systemic therapy and higher resolution imaging modalities,the incidence of pituitary metastases has increased over the past decade to 1-1.1% of all patients with brain metastases [1, 2]. The surgical management of PGM is complicated by the proximity of the metastasis to the adenohypophysis/neurohypophysis, cavernous sinus, optic apparatus, and internal carotid artery [5]. Local tumor management typically involves surgical excision or radiation therapy [1]. Due to anatomic constraints and tumor growth patterns, many patients’ undergoing surgical excision may suffer from increased morbidity or tumor recurrence with an approximate overall post-operative complication rate of 23.4%, CSF leak rate ranging from 7.11% to 12.8%, and hypopituitarism rate of 9.2% [1, 2, 5, 6].

Radiosurgery has been increasingly employed for benign and malignant brain tumors, including pituitary adenomas and brain metastasis [7–17]. Stereotactic radiosurgery has been shown to afford high rates of tumor control with minimal toxicity for pituitary microadenomas and macroadenomas [5, 10, 11, 13, 17–19].   It is therefore reasonable to consider SRS as a viable treatment option for patients with PGM.  To date, a limited number of studies have documented outcomes for patients with PGM treated with SRS.  We therefore sought to systematically review the currently available literature and provide an overall analysis of outcomes for patients undergoing SRS for pituitary metastases.

## Methods

### Search strategy

A systematic review was conducted in accordance with the Preferred Items Reporting in Systematic Reviews and Meta-analysis (PRISMA) guidelines [20]. The search phrase “(Stereotactic radiosurgery OR SRS OR radiosurgery) AND (Pituitary metastases)” was entered in the PubMed and Science Direct databases. Databases were searched from their inception through November 2025. No search filters were applied. We excluded systematic reviews/meta-analyses, case reports, conference abstracts, and technical notes. Two authors (T.K) and (N.H) conducted the searches independently with a third author (S.J) mediating discrepancies.

### Quality assessment

Quality assessment was performed using the Newcastle Ottawa scale with the threshold for inclusion set at > 6.

### Data collection

The following pre-defined variables were collected: study design, number of patients, median age, median prescription dose (Gy), median tumor volume (cm^3^), median optic apparatus dose (Gy), primary tumor histology, diabetes insipidus (DI) prior to SRS, cranial nerve deficits prior to SRS, and hypopituitarism prior to SRS (defined as biochemical evidence of any anterior or posterior pituitary hormone deficit excluding antidiuretic hormone as diabetes insipidus was documented separately). Furthermore, recovery of diabetes insipidus, hypopituitarism, or cranial nerve deficits was noted following SRS. Local tumor control rates and median overall survival noted from time of SRS to tumor progression or death respectively were collected. Upon completion, data was consolidated into a singular database and uploaded for statistical analysis.

### Statistical analysis

Continuous variables were analyzed using descriptive statistics and reported as median (range) or event rate (%). Median overall survival and follow up was calculated using the median of medians method as described by McGrath et al. [21]. Forest-plots displaying the pooled local control rates across the studies were generated using a random effects model with inverse variance weighting method adjusted using the method of Knapp-Hartung. Heterogeneity was assessed using the I^2^ statistic with an I^2^ of < 50%, 50%-75%, and > 75% representing low, moderate, and high heterogeneity respectively. Biologically equivalent doses were reporting assuming an α/β of 10. Studies not reporting the necessary data points were omitted from these analyses. Analysis was conducted using the publicly available “R” programming-based software developed by Fekete et al. [22].

## Results

### Literature search results

**Fig. 1:**
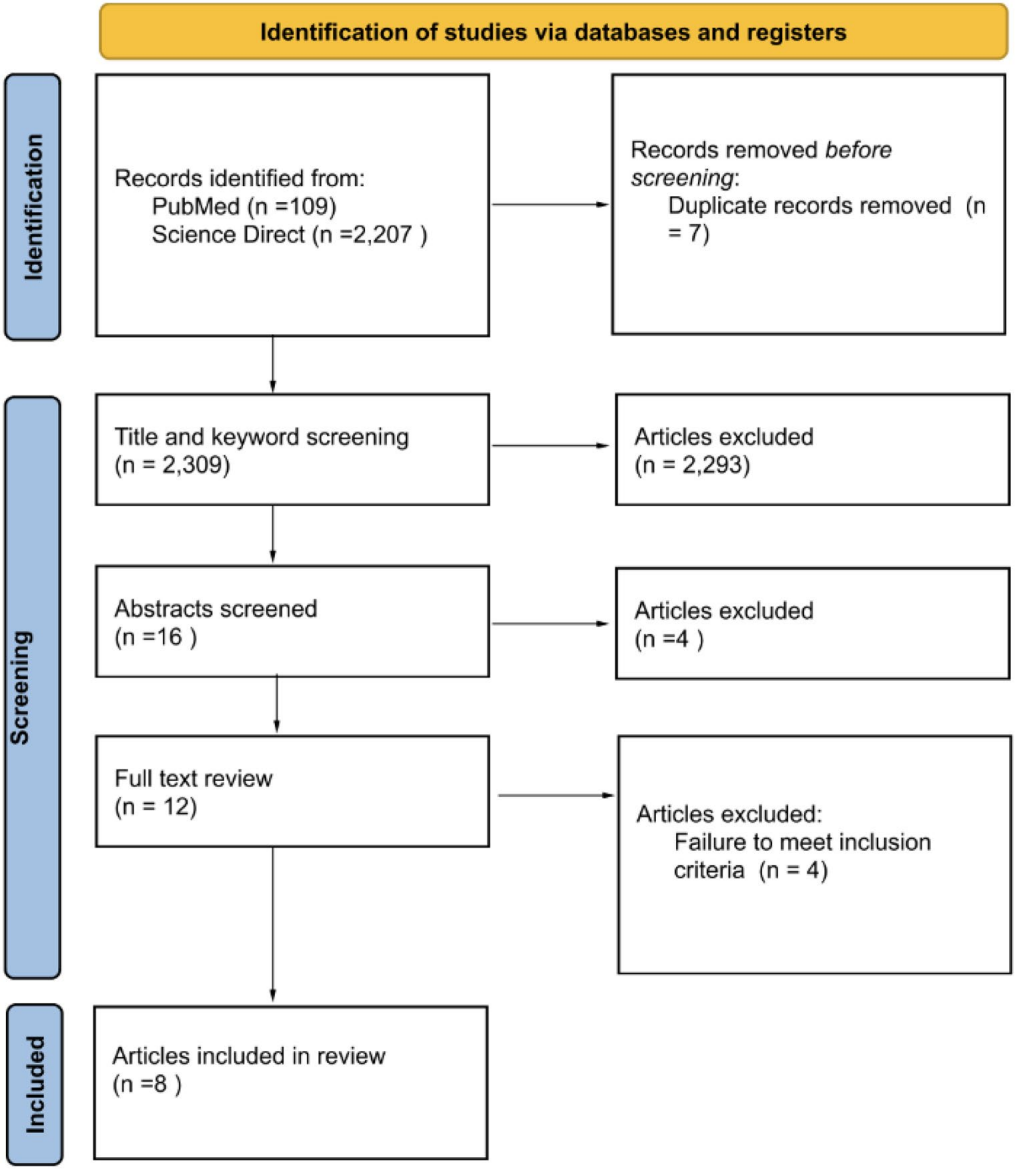
Literature review diagram based on the 2020 PRISMA guidelines

A PubMed and Science Direct search returned 109 and 2,207 results respectively for a total of 2,316 eligible articles. There were 7 duplicated articles across the two databases. Article titles and keywords were therefore assessed in 2,309 results with 2,293 undergoing exclusion. Sixteen articles underwent abstract screening with four being excluded at this stage. The total number of articles undergoing full text review was 12. From these 12 results, 4 articles failed to meet inclusion criteria. The final 8 articles underwent screening for quality assessment, with all 8 articles deemed appropriate for inclusion in the final analysis. A PRISMA flow diagram detailing the literature search is presented in Fig. 1.

### Study characteristics

Overall, 1 (12.5%) study was a multi-institutional retrospective design, while the remaining 7/8 (87.5%) were single center retrospective studies (Table 1). One hundred and nineteen patients with a median age of 62.0 years (R: 52.2–69.0) were treated with SRS. All patients had a single lesion. The most frequent primary tumor histology was lung cancer (50/119,42.0%), breast cancer (30/119,25.2%) and renal cell carcinoma (9/119,7.6%) (Table 1).

**Table 1:** Baseline study characteristics

Study	Study Design	Number of Patients	Median Age (range)	Primary tumor origin (n, %)	**Median Tumor volume (cm**^**3**^)	Median tumor diameter (cm, range)	Median Prescription Dose (Gy, range)	Median max dose to optic apparatus (Gy)
Kano et al.	Single institution retrospective	18	57.6 (27.0–81.1)	Lung 6 (33.3) Breast 4 (22.2) Melanoma 2 (11.1) Kidney 4 22.2) Gastric 1 (5.5) Colon 1 (5.5)	3.5 (0.2–18.0)	NR	13.0 (9–18) BED: 29.9 Gy	< 6 (3.8–13.3)
Chon et al.	Single institution retrospective	7	62.0 (40.0–64.0)	Lung 5 (71.4) Breast 2 (28.6)	1.5 (0.5–3.2)	1.7 (1.0–2.3)	31.0 (5fx) BED: 50.2	< 5
Benjamin et al.	Single institution prospective	5	69.0 (57.0–80.0)	Lung 3 (60.0) Breast 1 (33.3) Gastrointestinal 1 (33.3)	1.7 (0.25–15.9)	NR	13.0 (11–14) (1fx) BED: 29.9 Gy 18.0 (3fx) BED: 28.8	4.05 (1.95–9.30)
Mori et al.	Single institution retrospective	4	60.5 (34.0–70.0)	Renal 1 (25.0) Lung 1 (25.0) Thyroid 1 (25.0) Parotid 1 (25.0)	2.8 (1.6–8.7)	NR	12 (12–12.3) BED: 26.4 Gy	< 10
Yang et al.	Single institution retrospective	4	62.5 (39–72)	Breast 2 (50.0) Renal 1 (25.0) Melanoma 1 (25.0)	1.9 (0.6–1.9)	2.7 (0.8–8.5)	NR	NR
Iwai et al.	Single institution retrospective	7	64.0 (47.0–78.0)	Breast 2 (33.3) Lung 5 (83.3)	3.1 (0.2–9.6)	NR	12.0 (10–14) BED: 26.4 Gy	10 (8–10)
Lin et al.	Single institution retrospective	11	52.2 (30.3–83.6)	Lung 5 (45.5) Breast 3 (27.3) Renal 2 (18.2) Other 1 (9.1)	4.0 (0.13–41.92)	NR	15.0 (11.5–18.0) BED: 37.5 Gy	< 10
Abou-Al-Shaar et al.	Multi-institutional retrospective	63	62 (57–71)	Lung 25 (39.7) Breast 16 (25.4) Renal 5 (7.9) Prostate 6 (9.5) Colon 3 (4.8) Other 8 (12.7)	1.5 (0.7–3.6)	NR	13 (12–16) BED: 29.9 Gy	10.2 (8.2–12)

*Abbreviations:* NR: not reported, BED: biologically equivalent dose

The median tumor volume treated was 2.35 cm^3^ (R: 1.5–4.0) with a median tumor diameter of 2.2 cm (R: 1.7–2.7) and median prescription dose of 13.0 Gy (R: 12.0–18.0) corresponding to a biologically equivalent dose (BED) of 29.9 Gy (26.4–50.2). Of note a single patient received a prescription dose of 31 Gy in 5 fractions in the study by Chon et al. corresponding to a BED of 50.2 Gy [2]. The median maximum SRS dose delivered to the optic apparatus was ≤10 Gy in (6/7, 85.7%) studies that reported data for this parameter (Table 1).

### Intracranial disease presentation

Extracranial metastatic disease at the time of SRS was noted in 85 (71.4%) patients. Prior to SRS, DI was noted in 37 (31.0%) patients with hypopituitarism noted in 36 (30.3%) patients. Overall visual field deficits were reported in 32.8%, cranial nerve II deficits, and other cranial nerve deficits (III, IV, V, VI) were reported in 4.2% and 39.5% respectively (Table 2).

**Table 2:** Clinical status prior to SRS

Study	Diabetes Insipidus (n, %)	Hypopituitarism (n, %)	Visual field deficits (%)	CN II deficits (%)	Other CN deficits (III, IV, V, VI) (%)	Extracranial Disease (n, %)
Kano et al.	7 (38.8)	7 (38.8)	2 (11.1)	2 (11.1)	III 3 (16.6) IV 1 (5.5) VI 2 (11.1)	13 (72.2)
Chon et al.	7 (100.0)	4 (57.1)	3 (42.9)	0 (0.0)	0 (0.0%)	7 (100)
Benjamin et al.	0 (0.0)	1 (33.3)	1 (20.0)	0 (0.0)	III 1 (33.3)	1 (33.3)
Mori et al.	0 (0.0)	2 (50.0)	4 (100.0)	3 (75.0)	VI 1 (25.0)	NR
Yang et al.	0 (0.0)	1 (25.0)	1 (25.0)	0 (0.0)	0 (0.0)	2 (50.0)
Iwai et al.	2 (25.0)	2 (25.0)	3 (42.9)	0 (0.0)	III 2 (25.0) II-IV and VI 1 (14.0)	7 (100)
Lin et al.	5 (21.7)	7 (30.4)	7 (63.6)	0 (0.0)	III 2 (18.2) IV 1 (9.1) V 1 (9.1) VI 12 (18.2)	8 (34)
Abou-Al-Shaar et al.	16 (25.4)	12 (19.0)	18 (128.6)	0 (0.0)	III 11 (17.5) IV 2 (3.2) V 3 (4.8) VI 4 (6.3)	47 (74.6)

### Tumor control and survival

**Fig. 2:**
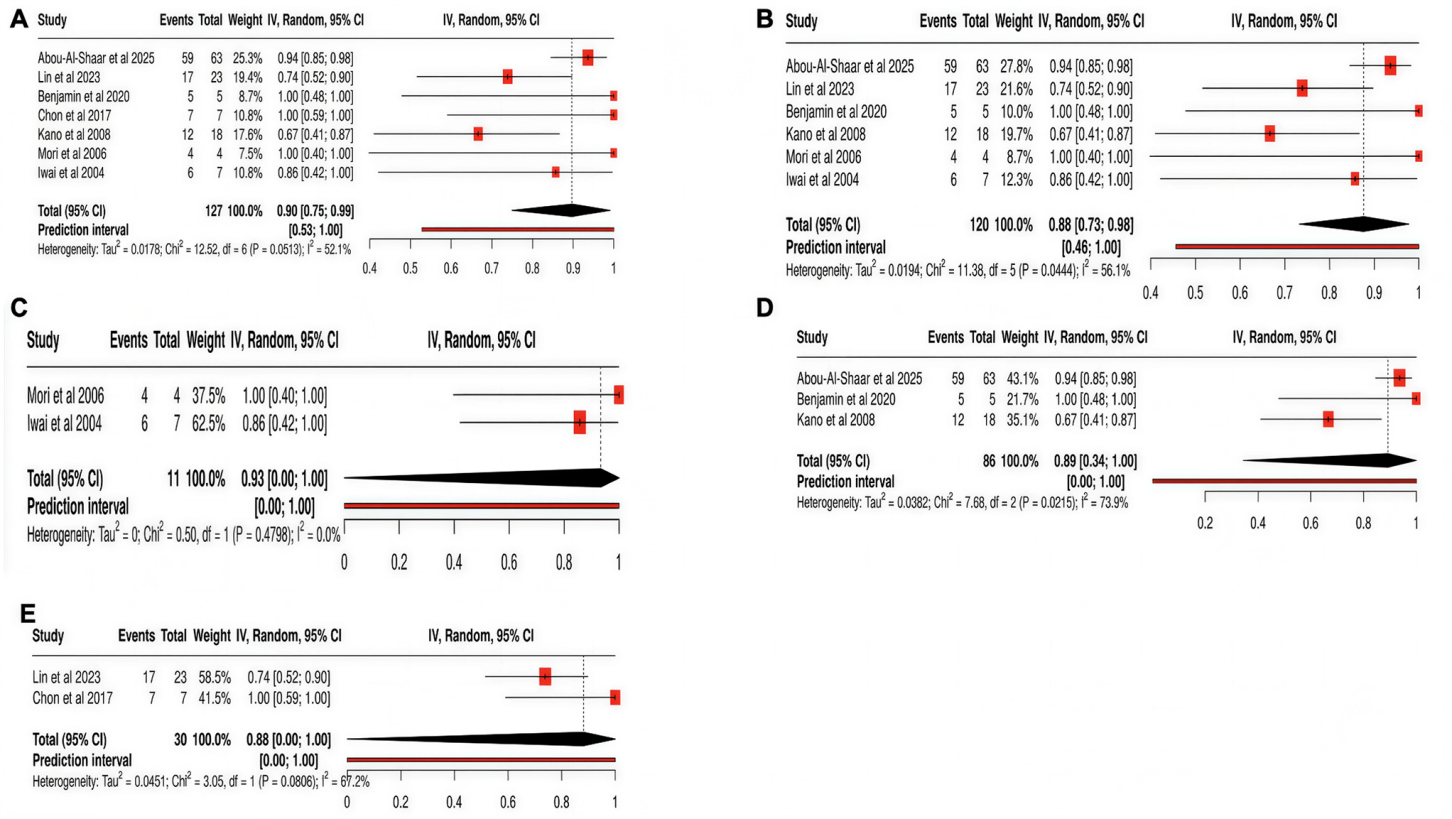
Forrest plot demonstrating a pooled estimation of the local control rate for pituitary metastases undergoing SRS. (**A**) Overall local control rates (**B**) Local control rates for single session SRS (**C**) Local control rates BED 26.4 Gy (**D**) BED 29.9 Gy (**E**) BED 37.5–50.2 Gy

Seven of the eight (87.5%) articles that were analyzed reported actuarial local control rates. Each individual rate was weighted according to sample size and synthesized into an overall estimated proportion demonstrated in Fig. 2. Across the literature, a 90% (95% CI: 75%-99%) (I^2^: 52.1%) actuarial local control rate was calculated (Fig. 2A). A pooled local control rate derived from studies reporting single session SRS rates was 88% (95% CI: 73%,98%) (I^2^: 56.1%) (Fig. 2B). A subgroup analysis of local control rates across a variable range of BEDs demonstrated local control rates of 93% (95% CI: NA-100%, I^2^: 0%), 89% (95% CI: 34%-100%, I^2^:73.9%) and 88% (95% CI: NA-100%, I^2^: 67.2%) for BED of 26.4 Gy, 29.9 Gy, and 37.5–50.2 Gy respectively (Figs. 2C, 2D, and 2E). The median follow-up across these seven studies was 7 months (R: 3.1–20.6). A pooled median survival rate across seven of eight reporting studies was 14 months (R: 5.2–30.0).

### Resolution of endocrine and cranial nerve symptoms

**Fig. 3:**
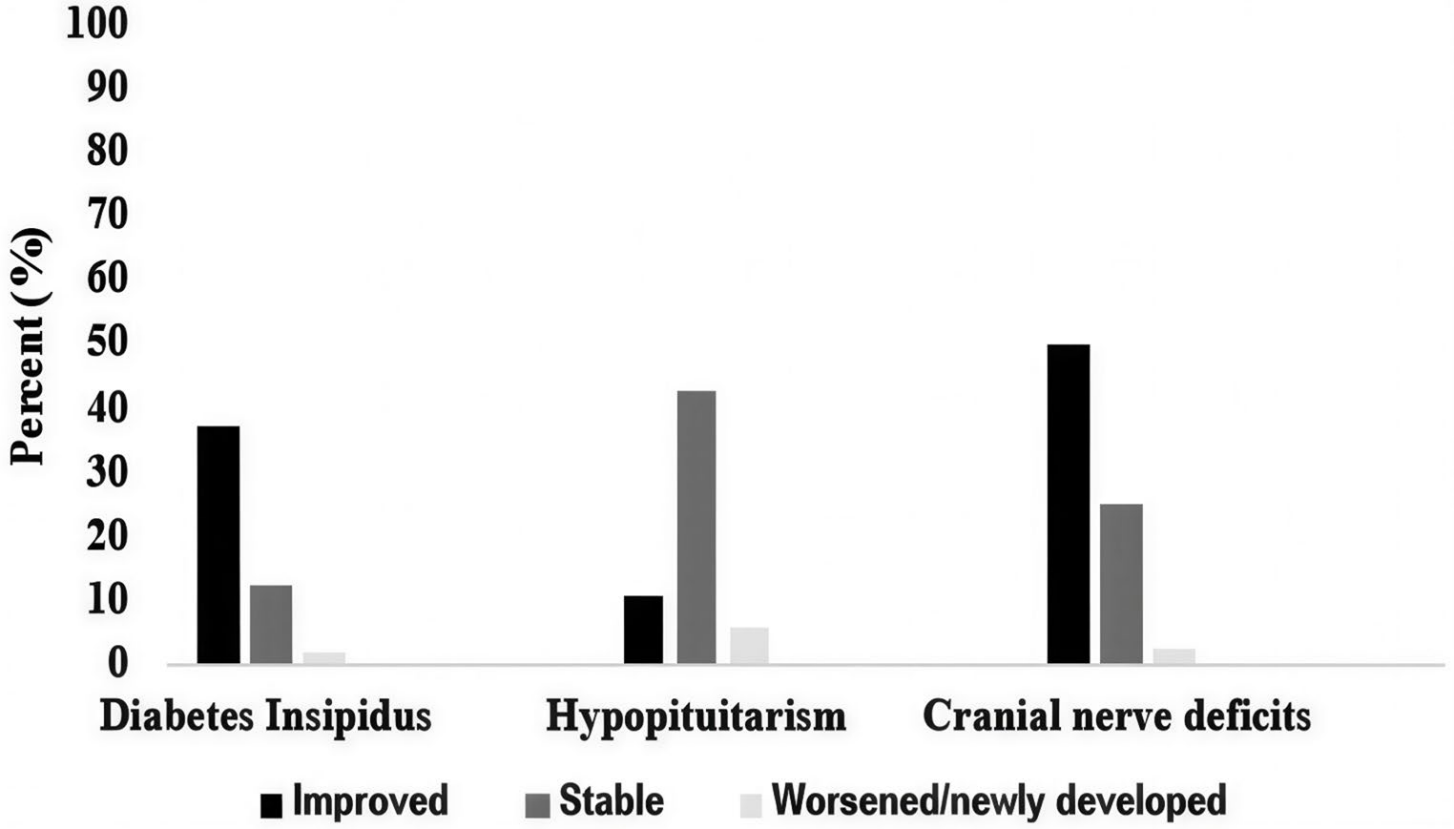
Endocrine and cranial nerve outcomes following SRS

Six of eight articles (75.0%) reported outcomes for cranial nerve and endocrine functional status following SRS. Diabetes Insipidus improved in 37.5%, was stable in 12.5%, and worsened/newly developed in 1.7% of patients (Fig. 3). Hypopituitarism improved in 10.7%, was stable in 42.9%, and worsened/newly developed in 6.0%. Cranial nerve deficits improved in 50.0%, were stable in 25.0%, and worsened/newly developed in 2.6% (Fig. 3).

## Discussion

With only eight studies available in the literature to date, reporting on SRS for the management of PGM is limited. Nonetheless, the existing literature demonstrates promising local tumor control results. Additionally, the development of cranial nerve deficits and new or worsening endocrinopathy is reasonably low. While the overall survival prognosis remains grim, the achievement of intracranial tumor control with SRS suggests that this strategy may be favorable for patients with advanced disease and poor performance status. Furthermore, in those who demonstrate radiographic features unfavorable for surgical excision, SRS can provide an alternative therapeutic strategy to mitigate potential excess surgical morbidity.

### Role of radiation in pituitary metastases

Radiation can be administered as either a definitive measure in treatment-naive lesions or adjunctively following surgery [1]. Advantages of definitive radiation treatment include diminished invasiveness and a reduced post-treatment recovery period [1]. Surgery in the pituitary/sellar region, especially in cases involving tumor growth to the cavernous sinus and skull base, carries notable surgical morbidity. While the incidence is low, life-threatening hemorrhage secondary to carotid injury is a major life-threatening risk associated with surgical excision for pituitary tumors [23]. Indeed, endoscopic endonasal skull base approaches have been associated with a population estimate 0–0.1% risk of ICA injury with the highest reported rate of 1% in a single cohort, a 7.11–12.8% risk of post-operative CSF leak, and 9.2% rate of new or worsening pituitary function [24–28]. This is a particular concern when tumor encasement of the internal carotid artery is noted and can be more frequent in metastatic disease which often exhibit disseminated growth patterns beyond the capsule of the pituitary gland [21]. While these are important treatment considerations, radiation therapy is not devoid of risk. Notably, radiation to the adjacent optic apparatus has created hesitancy among clinicians. Fortunately, this has received extensive study with a generally agreed upon single session dose of 8–10 Gy considered as a maximum safe dose for the optic apparatus [5, 29–32]. Across the reporting studies in this review, the majority adhered to a single dose of ≤ 10 Gy to the optic apparatus with only a 2.6% rate of new onset cranial nerve deficits following SRS. These findings re-demonstrate the safety of delivering radiation to the pituitary gland. It is important to note however, that the cases in this study had less optic nerve involvement and received lower doses to the visual apparatus than would be generally experienced by tumors with severe optic nerve compression. Ultimately, there may be an element of case selection across the study favoring smaller less invasive tumors, as larger more compressive tumors are not generally considered for SRS.

### Tumor control and survival benefits

In the largest study to date Al-shaar et al. demonstrated a 93.1% LC rate at 12 months of follow up [4]. Across the literature, we found a pooled LC rate of 90% [2–6, 23, 33]. Prescription doses > 15 Gy and administration of SRS < 2 months from symptom development have been associated with improved local control [6, 33]. However, Al-shaar et al. demonstrated a lower prescription dose threshold of > 10 Gy was sufficient for significantly improving progression free survival in a much larger cohort [4]. Interestingly we demonstrated that a median BED of 26.4 Gy had improved local control relative to BED 29.9 Gy and 37.5–50.2. Upon further examination the median tumor volumes between these groups was 2.95 cm^3^, 1.75 cm^3^, and 2.75 cm^3^ respectively. Additionally, the predominant primary tumor histology across all BED subgroups was lung. However, there existed substantial heterogeneity between groups with respect to non-lung primary tumor histology. Indeed, primary tumor histology is a well-established predictor of BMs responsiveness to SRS, and therefore could explain the subtle differences noted in local tumor control. Finally, overall heterogeneity across studies were noted in the BED ranges 29.9 Gy and 37.5–50.2 Gy which limits comparison. As previously discussed, achieving tumor control with the minimum effective radiation dose is critical for lesions in the pituitary gland.

Unfortunately, most patients with PGMs experience death secondary to systemic disease progression [4]. Typically, this patient population presents with active systemic disease in over 50% of cases at the time of diagnosis [6, 34–37]. Indeed, in our review we identified 64.8% of patients with extracranial disease at the time of the intracranial lesion diagnosis. When analyzing differences in median overall survival rates across studies reporting < 50% and > 50% extracranial disease involvement at the time of SRS rates of 16.0 and 12.7 months were obtained. Consequently, achieving intracranial disease control is only a part of an overarching multidisciplinary effort. Other predictors of diminished survival reported in the literature include: multiple metastases, advanced age, and a short interval between primary and PGM diagnosis [6].

### Symptom control and physiological function restoration

A large proportion of individuals in this population present with some degree of pituitary dysfunction, most commonly diabetes insipidus and optic/oculomotor nerve deficits [1, 6]. We identified 31.0% of patients presenting with diabetes insipidus, 30.3% with hypopituitarism, and 51.3% with a cranial nerve deficit prior to SRS. In the setting of an overall poor prognosis, SRS can provide a palliative benefit for symptomatic tumors. Fortunately, hormone replacement therapy offers good control of endocrine symptoms while regression of PGMs following SRS mitigate symptoms related to mass effect [6]. Nonetheless, irradiation of the sella carries a risk of injury to the optic apparatus and hypopituitarism [4, 33]. One implication of these findings is the necessity of long-term endocrine/opthalmological follow up following SRS. The results of the pooled analysis were encouraging demonstrating only a 6% rate of newly developed or worsening hypopituitarism and 2.6% rate of newly developed cranial nerve deficits following SRS. On the issue of post-radiation cranial nerve deficits, it is critical to distinguish between radiation toxicity or continued tumor progression associated with compression. Kano et al. attributed 2/3 (66.7%) cases of newly developed cranial nerve deficits following SRS to true tumor progression and 1/3 (33.3%) to radiation toxicity [6].

### Risk of optic chiasm irradiation

Historically, the optic apparatus has been a limiting factor in the implementation of radiosurgery to the pituitary gland [1]. Generally, a safe distance from target volume to the optic chiasm must be measured prior to the decision to proceed with SRS [1]. Fractionated radiosurgery is a potential solution in situations in which the cumulative dose distributed to the optic apparatus is a concern [1]. Fractionated SRS schemes provide similar LC rates with reduced adverse events and can consistently limit radiation to the optic apparatus [2, 3]. In a series of 7 pituitary metastases treated with 31 Gy over 5 fractions (BED: 18.0 Gy), a 100% local control rate with 100% resolution of diabetes insipidus and cranial nerve deficits following SRS was reported [2]. Furthermore, this study demonstrated no anterior or posterior pituitary dysfunction following SRS [2]. The fractionated approach may play an important role especially in lesions with aggressive growth patterns and those abutting cranial nerves where the radiation toxicity risk is theoretically amplified. Currently only two of the studies included reported on outcomes of patients undergoing fractionated SRS [2, 3]. Future work ought to examine toxicity related outcomes across single and fractionated PGM SRS cohorts.

### Stereotactic radiosurgery candidacy

Patients with smaller sellar PGM without progressive visual loss or patients who are not surgical candidates have been treated with SRS [1]. As PGM often occur in the setting of advanced disease, the decision to proceed with an intent of surgical excision of a symptomatic PGM needs to be balanced with the patient’s overall disease course and available systemic treatment options [3]. The ability to deliver SRS with concurrent systemic therapy in this disease setting is advantageous. While not always the case, intervals of systemic therapy cessation may be necessary around the time of conventional radiation therapy due to toxicity concerns and surgical excision to increase operative fitness. As a result, the decision to proceed with SRS for PGM should be made in a multidisciplinary fashion with input from neurosurgery, radiation oncology, medical oncology, endocrinology, and neuro-ophthalmology.

## Conclusion

Stereotactic radiosurgery appears efficacious for PGM. Local tumor control rates are excellent, and many patients recover from pre-operative hormonal/cranial nerve deficits. Despite this, concurrent extracranial metastatic disease is common and presents a barrier to prolonged survival. Intensive multidisciplinary management alongside SRS in select patients may optimize outcomes in patients with PGM.

## Data Availability

Data is available upon reasonable request to the corresponding author.
